# A qualitative study of the government’s engagement of the private health sector in the delivery of Ghana’s COVID-19 emergency response

**DOI:** 10.1136/bmjgh-2023-014217

**Published:** 2024-12-18

**Authors:** Gordon Abekah-Nkrumah, Patience Aseweh Abor, Kingsley Addai Frimpong, Maureen M Martey, Sofonias Getachew Asrat, Francis Chisaka Kasolo

**Affiliations:** 1 University of Ghana Business School, Accra, Ghana; 2 World Health Organization, Accra, Ghana; 3 Private Sector and Bilateral Engagement, Ghana Ministry of Health, Accra, Ghana; 4 World Health Organisation, Accra, Ghana

**Keywords:** COVID-19, Health policy, Health systems, Qualitative study

## Abstract

**Introduction:**

There is a growing literature on the significance of private sector engagement and collaboration for optimal response to health emergencies. The current study examines how the private sector was engaged by the Ghanaian government to implement effectively the national COVID-19 emergency response.

**Methods:**

The study drew on a qualitative research design, interviewing 20 respondents in 15 unique organisations. Interviews were recorded, transcribed and analysed using a thematic analytical approach.

**Findings:**

The findings of the study suggest that the government demonstrated leadership in mobilising, resourcing, and collaborating with the private health sector to deliver its pandemic response via a defined emergency response plan, a coordinated pandemic response structure and a robust platform for information gathering and sharing. However, the government fell short of providing the enabling environment for the private health sector to expand their capacity to meet increased demand for health services during the pandemic. There were also challenges related to the over concentration of resources in the public health response and national level structures to the detriment of clinical care and sub-national level structures. Generally, the findings also indicate a fragmented private health sector that is not only unattractive for the government to engage and collaborate with, but also weak in terms of capacity (financial and human resources) to partner government and respond to any major health emergency.

**Conclusion:**

There is a need for policymakers to put in place an appropriate policy framework that will help in organising, engaging and collaborating with private health entities. The gaps identified and lessons learnt from implementing the pandemic response should be addressed as a matter of urgency to improve the readiness of Ghana’s health system for future health emergencies.

**WHAT IS ALREADY KNOWN ON THIS TOPIC**

- Results from studies in both high and low–middle income countries indicate that collaboration between the private and public health sector was crucial in responding to the COVOD-19 pandemic, although evidence regarding this is scanty in Ghana.

**WHAT THIS STUDY ADDS**

- High levels of fragmentation within the private sector coupled with weak capacity rendered collaboration efforts very challenging.
- Notwithstanding the challenge, government through the Ministry of Health showed great leadership (mobilisation of needed resources, creating the right environment and structures) that galvanised and incentivised a proactive private sector to collaborate with the public sector in responding to the COVID-19 pandemic.

**HOW THIS STUDY MIGHT AFFECT RESEARCH, PRACTICE OR POLICY**

- There is the need for further research not only to understand the role of the private health sector (important or otherwise) in optimal health services delivery, but also strategies via which the public health sector can collaborate with their private counterparts.
- For many low- and middle-income countries such as Ghana, the COVID-19 response (resource mobilisation strategies, incentives, structures and leadership) can constitute a template that can be improved on and used to better mobilise and engage the private health sector for optimal health services delivery both in normal and emergency periods.

## Introduction

 Both high and low–middle income countries had major challenges in implementing their respective COVID-19 emergency response plans. In a few countries (eg, Republic of Korea, Singapore, New Zealand, etc) where implementation results were relatively better, collaborative governance (a system where government works jointly with other stakeholders to produce public goods) and, in particular, private sector collaboration was identified to be one of several factors explaining success.^1^,^4^ Specifically, collaboration between government and the private sector in research and development of vaccines, provision of personal protective equipment (PPE) and other medical equipment such as ventilators, building of healthcare infrastructure, mobilisation of skills and funds, development of software applications for surveillance and monitoring of COVID-19, as well as enhancing testing and laboratory capacity was a critical factor in response capacity.^1 3 5^

In the Ghanaian context, the COVID-19 pandemic and response were concentrated in urbanised regions where there is a higher concentration of private health sector resources. For example, the private health sector was responsible for 51.7% of health consultations in urban centres in 2019 compared with 45.7% in public health facilities.^6^ In Accra (the capital city and epicentre of COVID-19 infections in Ghana), the private health sector represented 68.8% of all health consultations in 2019 compared with 31.3% in public health facilities, in addition to eight out of every 10 health facilities being private.^7^ Also, the majority of operators in Ghana’s pharmaceutical (80%) and medical laboratory (23 of the 33 highly recommended) sub-sector are private, and based in Accra and Kumasi, the two most populous cities in Ghana.^8 9^

In contrast, critical care infrastructure (82 staffed intensive care unit (ICU) beds and 62 ventilators in public health facilities compared with four staffed ICU beds and five ventilators in private health facilities) was concentrated in public health facilities before the COVID-19 pandemic.^10^ This necessitated greater collaboration between the government and the private health sector through governance of the whole health system to respond to the COVID-19 pandemic. Apart from a study that looked at the role of the private health sector in the early stages of the COVID-19 pandemic in Ghana, Nigeria and Bangladesh,^11^ there is currently little or no information on how the government of Ghana engaged and collaborated with the private health sector in the planning and delivery of the COVID-19 emergency response. Thus, the aim of this study is to examine the engagement of the private health sector by the government in the delivery of Ghana’s COVID-19 pandemic response. The private health sector is defined to include all ‘individuals and organisations that are neither owned nor directly controlled by governments and are involved in the provision of health services’.^12^ It can be classified into sub-categories such as for-profit and not-for-profit, formal and informal, domestic and international.^12^ In the context of Ghana, this will include faith-based providers, for-profit providers and not-for-profit providers within the non-governmental and civil society space. For the purposes of placing the results of the study in context, it is important to emphasise that there is currently little or no integration of the public health sector with the private health sector. In many instances, interaction happens mainly at the level of regulation as systems for human resources, finance, technology, service delivery, etc are disparate.

### Conceptual framework

The paper adopted the WHO governance behaviours as a conceptual framework (see table 1). The governance behaviours were conceptualised as part of the WHO strategy ‘Engaging the private health service delivery sector through governance in mixed health systems’.^13^ Launched in 2020, this strategy contributes a specific focus on the private sector as part of health systems governance and systems strengthening. The governance behaviours are elaborated in table 1 alongside key questions explored throughout the paper.

**Table 1:** Governance behaviour research framework

Governance behaviour	Research question
Deliver strategy. Government establishes the priorities, principles, and values for the health system, and works out how to translate these priorities, principles and values into practice	How was the COVID-19 response plan and strategy defined and what processes informed this?
Align structures. Government takes the required actions to align public and private structures, processes and institutional architecture	How were public and private structures aligned and adapted for the COVID-19 response?
Build understanding. Government facilitates information-gathering and sharing about all elements of service provision in the health system	What were the systems for data capture and how did these facilitate or hinder information exchange for the COVID-19 response?
Enable stakeholders. Government authorise and incentivise health system stakeholders to align their activities and further leverage their capacities, for national health goals	How were financing and regulatory systems deployed for the COVID-19 response?
Foster relations. Government establishes mechanisms that allow all the relevant stakeholders to participate in policymaking and planning	What coordination arrangements were established and how did these facilitate or hinder sectoral engagement for the COVID-19 response?
Nurture trust. Government leads the establishment of transparent, accountable and inclusive institutions at all levels to build trust	Did the COVID-19 response instil sectoral and consumer trust in the health system?

## Methods

The study was informed by key informant interviews of 20 individuals from 15 unique organisations purposefully selected and in some instances through snowballing. Respondents were drawn from development partners (five), public sector agencies (six), private not-for-profit actors (four), private for profit actors (two) and quasi government actors (three). Admittedly, 20 individuals may be considered small for a diverse sector such as health. However, the focus was not just on the number of people to interview but the number of unique agencies, in addition to those we believed have the information needed. Most importantly, by the time we reached the 20th interviewee, we realised a certain level of saturation, hence concluding that extra interviews may not yield additional insights. The interviews lasted approximately 1 hour per respondent. All interviews were done online via the Zoom conferencing software, with the exception of one respondent who requested a face-to-face meeting. With the permission of respondents, all interviews were recorded and transcribed on a real-time basis via the use of Otter.ai (Otter.ai Inc, Mountain View, CA, USA), an artificial intelligence software with the capacity to record and transcribe at the same time.^14^ The voice-recorded interviews were later cross-checked with the real-time transcription done by Otter.ai to correct any anomalies in the transcripts.

A thematic approach was used to analyse information contained in the transcripts. To ensure that the analysis was consistent with the governance behaviours, an Excel template was developed with columns for each governance behaviour as well as sub-questions to capture the content of the transcripts. The content of each transcript was inserted in the relevant columns in the Excel template. Emerging themes from the Excel template across different respondents were documented and later used as the basis for presenting the findings of the study. The findings are structured in line with the governance behaviours conceptual framework.

In line with standard protocols between the Ministry of Health (MOH) and the WHO country office in Ghana, the study was granted administrative approval from the MOH. This approval formed the basis for contacting respondents for data collection. In addition, informed consent was sought from respondents before interviews were conducted.

## Results

### Deliver strategy. How was the COVID-19 response plan and strategy defined and what processes informed this?

A Private Sector Unit exists within the Policy Planning, Monitoring and Evaluation (PPME) Directorate of MOH, although its functions were recently incorporated into the new Bilateral and Domestic Resource Mobilisation Unit of the PPME. In addition, a private sector policy^15^ exists but was barely implemented, in addition to the fact that many respondents were not familiar with the existence of the plan as below.

Yes, I am aware there is a private sector desk and also a private sector policy from the MOH, but I have not seen that before and I cannot tell whether the private sector was engaged in the development of that policy. *Private Sector Official (R4)*

Respondents indicated that a revised version of an emergency response plan (National Strategic COVID-19 Response Plan (NSCRP) for the period July 2020–December 2024) was prepared by the government to guide Ghana’s pandemic response. Stakeholders from the private sector, however, did not feel that they were adequately engaged in developing the NSCRP. This meant that they had little information both on the development and revision of the NSCRP and on how lessons learnt from implementing the first version were incorporated in the revised version.

I know of the existence of the COVID-19 emergency response strategy for Ghana. I must say that the development of that document was at the level of the presidency and so we were not involved in it. *Private Sector Official (R4)*

### Align structures. How were public and private structures aligned and adapted for the COVID-19 response?

The findings indicate that the government realised at the peak of the first and second waves that public facilities alone would not have been enough to respond to the surge in cases. Thus, to bridge the gap and respond adequately, the government worked with the private health sector first on testing. This meant the development of testing guidelines, training of private laboratories which had the capacity to test and were willing to do so, and finally accrediting them to offer testing services. This helped to increase the national testing capacity substantially.

In a similar fashion, selected private health facilities were trained by the Ghana Health Service (GHS)^16^ to manage COVID-19 cases at their facilities, while hotels and faith-based organisations provided spaces for quarantine and isolation. With the exception of mPharma (a private pharmaceutical company), with whom the government signed a memorandum of understanding to supply COVID-19 vaccines, and a few private pharmacies that participated in the vaccination exercise, Ghana’s vaccination strategy was predominantly carried out by public health facilities. Respondents believed that unlike public health, less attention was paid to clinical care in emergency situations before the COVID-19 pandemic.

Responses from the interviews suggested that some stakeholders in the health sector took advantage of challenges encountered at the early stages of the pandemic to derive personal benefits. Anecdotal evidence from respondents (from public and private actors) suggests that some private sector actors charged exorbitant prices for laboratory tests, while in some instances health facilities were not truthful about their bed situation.

Non-public facilities were not interested in getting into testing because of the thinking that it is hazardous. However, when some of us started, other small ones also got in there, amidst reports of unconventional practices such as positive results being negative and all that. In the area of case management, the main issue was with health providers not being truthful about their bed capacity and sometimes rejecting patients because of the risk. *Private Sector Official (R10)*

### Build understanding. What were the systems for data capture and how did these facilitate or hinder information exchange for the COVID-19 response?

Before the implementation of the COVID-19 emergency response plan, the public health data repository did not have adequate and reliable data on services provided by the private sector, given that only a few private health facilities (note: there is currently no official statistics on level of accuracy and quantum of data sent from the private sector to the public health data repository) were entering data into the public health data repository referred to in Ghana as the District Health Information Management System. Thus, service output data relied on for decision-making at the national level was not reflective of total output within the health sector.

The implementation of the COVID-19 emergency response plan provided an opportunity to transmit data from the private health sector and tertiary hospitals to the MOH, responsible for all health-related data. This was done through the development and deployment of a Surveillance Outbreak Response Management and Analysis System (SORMAS) to collect data on testing and confirmation of cases, validation of transition from positive to negative status, case management and vaccination of citizens. Data collection happened at the district level and was aggregated at the national level through the Emergency Operations Centre to the National Technical Coordinating Committee (NTCC) and finally the Inter-Ministerial Committee. The MOH and GHS trained private sector entities on data capture and in some instances made available equipment and data entry assistants to them.

The private sector was adequately trained to be able to report data and in some instances the GHS and MOH sent people and equipment to private providers. Data capture and reporting was mainly from the private to the public but the private did not get to know what was happening in the public space. *Development Partner Official (R15)*

Responses from the interviews suggest that the information collected constituted the basis for decisions made, especially by the national government. These include presidential addresses and directives. There is, however, scanty evidence that data were used at operational levels of the response or across the health sector.

### Foster relations. What coordination arrangements were established and how did these facilitate or hinder sectoral engagement for the COVID-19 response?

With the exception of health facilities under the Christian Health Association of Ghana (CHAG), the private for-profit sector does not always have the required structures for meaningful engagement with the government. Those advocating for the interest of the different actors in the private sector keep changing, beside the fact that there are a multiplicity of private sector groups that are all pushing to have direct engagement with MOH, making it difficult for sustained engagement between the private health sector and government. As a result, the majority of the private health sector was excluded from the communication and coordination structures.

While SORMAS became the major digital tool for collecting and communicating data and information from lower levels to higher levels, it was nonetheless controlled by the GHS, a public agency, thus restricting access by the private health sector, who were equally required to report to MOH through the SORMAS. Also, information flow and the pandemic response coordination structure was built around public sector actors. Thus, there was no space within the structure to intentionally and systematically involve the private health sector or monitor their activities. In lieu, the private sector advocated with policymakers for space within the coordination structure and relied on social media, such as WhatsApp groups, to communicate and coordinate, especially at the task team level.

### Enable stakeholders. How were financing and regulatory systems deployed for the COVID-19 response?

The government introduced a raft of financial measures specifically aimed at incentivising health facilities and their staff to enable them to implement the COVID-19 response plan. This included insurance cover, 3 months tax waiver on salaries and 50% of basic salary to be paid as allowance to all frontline healthcare staff. Respondents indicated, however, that only frontline healthcare staff in public health facilities benefited from the tax waiver.

At the health facility level, the government distributed free PPE to all health facilities, and equipment mainly to public and faith-based health facilities under the CHAG. The government also established financial stimulus packages to help the private sector in general to recover from the shock of the pandemic and promote growth,^17^ but did not target the private health sector in particular. This made it difficult for private health entities to secure funds to expand their operations in response to the demand for health services. Some respondents noted that emphasis was placed on guidelines and protocols that were used by both the private and public health facilities to deliver services for the COVID-19 response and other public health emergencies. However, appropriate incentives and systems to help the private health sector to improve its capacity to deliver services was almost absent.

### Nurture trust. Did the COVID-19 response instil sectoral and consumer trust in the health system?

Inequities in the distribution of incentives and equipment between sectors were prevalent during the response. Leaders from private health facilities complained that even though the government at the onset of the response announced an incentive package for all frontline health facility staff, those in the private sector were not catered for. Additionally, public health facilities received supplementary equipment to help them respond appropriately to the pandemic, but the private sector was left out of this package. They regarded this as unfair treatment given that they rendered service in support of the government’s effort.

Yes, people may talk about the fact that we are for profit, but the fact is also that our absence in a time like this and even in normal times can be catastrophic for the nation since we provide such an essential service to the Ghanaian public. *Quasi-Government Health Facility Official (R 10)*

## Discussions

The findings of the study suggest that although the private health sector in Ghana operated in a constrained environment and lacked a strong capacity to support the government in a pandemic situation, it demonstrated proactiveness and a willingness to work with the government under existing constraints to deliver the COVID-19 emergency response.

The study results indicate that the current state of engagement with the private sector is generally weak, as indicated by a less visible organisational arrangement (downgraded Private Sector Unit in the MOH), with an outdated policy for private sector collaboration. This means that the private health sector is currently not aware of the rules of engagement, and the role expected of it. This is inconsistent with prescriptions of existing literature on collaborative public–private delivery of health services.^13 18^ However, given the proactive role played by the private health sector even in the mist of existing constraints, there is the potential to create an enabling environment with robust institutional structures.

Additionally, the findings suggest that although the response structures at the national level were far better than those at the regional and district levels, these were generally robust and well-coordinated. However, the lack of collaborative structures between the private and public sectors meant that providers in the public sector had to take more responsibility than it was capable of, leading to dysfunctional and opportunistic behaviours in both sectors. Notwithstanding the constraints, and in line with existing evidence,^1 3 5 19 20^ the private sector demonstrated a great willingness to support the effort of government by proactively and speedily recalibrating its systems and structures to augment the efforts of the government to deliver the pandemic response.

The collaborative and responsive data governance atmosphere created by the MOH as part of the pandemic response strategy is noteworthy. This allowed for harmonisation of data capture and reporting, and can be explained by three factors: (1) the importance of data in national level decision-making during the pandemic, which was fluid and evolved over time; (2) ownership of the COVID-19 data repository (SORMAS) by the NTCC through the MOH, which was important in ensuring that relevant stakeholders reported data into the system; and (3) the process leading to the decision to report data into the SORMAS was inclusive, coupled with adequate support (eg, training of staff and supply of equipment) to both private and public sector providers to make it relatively easy for them to enter data into the SORMAS. Thus, the implementation of the COVID-19 response has provided lessons on the importance of sectoral leadership and support in creating a collaborative and inclusive data governance system for the health sector.

A key issue that needs the attention of policymakers in the context of fostering relations and nurturing trust is the issue of citizens’ inability to access private health facilities during the peak of the pandemic when public health facilities were full. This may be an indication of poor collaboration between the private and public sectors as well as below optimal contingency planning. A strong contingency planning capability would have made it possible for the MOH to identify private sector entities with the capacity to augment the government’s effort and negotiate with them and make it possible for citizens to access their services without the challenge of paying at the point of use, as was the case in South Africa and Peru, Philippines, Morocco and Mexico.^21^,^24^ Related to this is the inequity in the distribution of incentives to frontline workers in a manner that disadvantaged the private health sector. Private sector entities filled a vacuum for essential health services that was created by the focus of the public sector on the response. Thus, incentivising the private health sector to augment the effort of government is clearly in the national interest.

Generally, the government’s effort at enabling stakeholders in responding to the pandemic was mixed. Inadequate support from government to the private health sector at a time when their businesses were directly hit by the pandemic made it difficult for them to expand their capacity to support the government. It is therefore not enough for policymakers to ask the private health sector to ‘step-up’ to challenges, but more importantly, they should empower the private health sector with the capacity to help in times of emergencies such as COVID-19. In this regard there may be the need for specific financial arrangements that target the private health sector to improve their capacity to respond to the demand for care, much more effectively than was done.

Finally, the findings of the study have clearly articulated the challenges of the private health sector, key among them being inadequate capacity and constrained access to low cost financing. These have potential negative implications for the ability of the private health sector to play its role both in normal and emergency situations. It will therefore be important for government to help organise the private sector into a coherent unit that can work with government and develop partners to improve capacity and to access low cost financing.

### Study limitation

Given that the COVID-19 response was concentrated mostly in urban centres, particularly Accra and Kumasi, the interviews focused on respondents mostly in Accra but with oversight responsibilities in the rest of the country. It is therefore possible that nuanced information at lower levels that may be crucial for public–private health sector collaboration and engagement may not have been captured. Thus, the results of the study should be interpreted with this limitation. However, this limitation does not invalidate the findings of the study since almost all policy decisions and major actions on the COVID-19 response were initiated and implemented in Accra.

## Conclusion

The findings provide clear lessons that are not only important for improving Ghana’s preparedness for managing a national level emergency, but also for better positioning the national health system to deliver on its mandate. A key lesson is the need for an appropriate policy framework that will help in organising and properly engaging and collaborating with all of the private sector (including non-traditional actors such as churches and mosques), that were key in providing not only resources (spaces for quarantine) but also serving as key channels to reach citizens with livelihood support and behaviour change messages.

The results equally emphasise the need for a more robust emergency planning system with the capacity to identify all relevant stakeholders (including the private sector), their roles, capacities and resources (eg, funds, personnel, equipment, training, etc) needed. This will serve to reduce constraints in the supply of services, promote access to services, reduce inequities, promote trust and therefore rally all citizens behind the state in responding to an emergency situation. The pandemic response has also brought to the fore health systems capacity challenges, especially in the private sector. This includes over concentration of capacity and investments in public health to the neglect of clinical care, and over concentration of efforts and resources at the national level to the detriment of sub-national level structures. The COVID-19 pandemic is a reminder that in a public health emergency clinical systems will play a major role in an effective response; therefore, there is the need to invest in clinical care systems in much the same way as public health systems, both at the national and sub-national levels.

An even more important lesson is the demonstration of leadership and ownership by the MOH in mobilising all stakeholders both in allocating resources and sharing data for critical decision-making during the pandemic. The politics of data governance in Ghana’s health system has been a major challenge for many years. However, the pandemic created the necessary conditions for the MOH to negotiate with and support stakeholders through the provision of equipment, training and personnel for the purposes of capturing and sharing data through the SORMAS. This success story can be leveraged to create a health sector-wide solution to address the existing data governance challenge. Finally, the health sector, through the leadership of the MOH, also demonstrated enough agility in mobilising stakeholders to develop the required protocols and guidelines to open up spaces and possibilities for private sector participation. This is indeed commendable as it is at variance with the characterisation of private health actors as only being interested in profit, hence the calls to regulate strictly their activities.

Overall, the pandemic response provides a great opportunity to address challenges identified as well as leverage on opportunities unearthed to develop a robust and resilient health system not only for responding to future emergencies but also for responding to the everyday health needs of citizens.

## Data Availability

All data relevant to the study are included in the article or uploaded as supplementary information.
